# Evidence on information, access frictions, and experience in public healthcare use in rural Bangladesh: a cluster-randomized field experiment

**DOI:** 10.1093/heapol/czag109

**Published:** 2026-09-07

**Authors:** Mohammad Asif Hasan Khan

**Affiliations:** Department of Economics, Skidmore College, 815 North Broadway, Saratoga Springs, NY 12866, United States

**Keywords:** public healthcare, demand-side frictions, credence goods, field experiment, informal providers, Bangladesh

## Abstract

In rural Bangladesh, many households continue to rely on private and informal providers even when public healthcare options are available at low or zero monetary cost. This paper studies whether the barrier is mainly misinformation, access frictions, or trust and uncertainty about public care. A cluster-randomized field experiment was conducted with 2320 households across 580 neighborhoods and three sequential treatment arms. Information provision sharply increases awareness of public care options but has limited effects on actual utilization. In contrast, encouraging a one-time experience with public care produces significant increases in public uptake, substitution away from informal providers, and meaningful reductions in out-of-pocket spending, especially on medicines. Households induced to use public care reduced total health spending by ∼601 Bangladeshi taka (USD 5.46 at 2023 exchange rate; 32% of the control group mean) relative to nonusers (95% CI 373–829 taka; *P* < 0.001), with most of the reduction driven by lower medicine expenditure. The findings suggest that improving beliefs alone is insufficient and that lowering uncertainty through a first successful experience is a key lever for shifting healthcare demand toward public options.

Key messages

- In rural Bangladesh, information campaigns substantially raised awareness of free public healthcare services but produced no change in actual provider choice. The results are consistent with households placing high value on familiarity, convenience, cost, and personal trust in existing informal providers, rather than lacking information about public alternatives.
- A single facilitated experience with public care—one successful consultation—increased uptake by 18 percentage points and reduced total household health spending by 32% (∼USD 5.46 at 2023 exchange rate), driven by lower medicine expenditure and reduced exposure to overprescribing informal providers.
- The findings are consistent with healthcare functioning as both a credence good and an experience good: information alone cannot overcome habitual provider loyalty when patients have no direct basis for comparing public and informal care quality.
- Low-cost experience-facilitation components—priority tokens, guided first visits, or accompanied facility referrals—may generate substantially larger returns than information campaigns and deserve greater attention in the design of primary care scale-up programs in low and middle income countries (LMICs).

## Introduction

In many low-income settings, households routinely seek medical advice from informal drug sellers even when certified health professionals are formally available. This pattern remains especially common in rural Bangladesh (Banerjee et al. 2004, Das et al. 2008, WHO and World Bank 2017). Informal providers frequently recommend excessive or inappropriate treatments, particularly medicines (Emons 1997, Das et al. 2016), contributing to high out-of-pocket costs and to antimicrobial resistance, now a major global public health concern.

Bangladesh has expanded several public healthcare delivery mechanisms in recent years, including community health clinics, government-run public facilities, and mobile health services. Despite these efforts, utilization of formal public healthcare, particularly in-person visits to public facilities, remains strikingly low in rural areas. This paper focuses specifically on barriers to in-person public healthcare utilization and asks which demand-side interventions can effectively shift households away from informal providers toward qualified public care.

Using a large-scale cluster-randomized controlled trial, I test three sequential, demand-side interventions: (i) information provision, (ii) practical access support, and (iii) encouragement to obtain a one-time assisted first use. The design allows me to isolate which constraint is binding and whether direct experience plays a distinct role.

The persistence of informal provider use despite available and often free public alternatives is well documented across South Asia and sub-Saharan Africa. In rural Bangladesh, Chaudhury et al. (2006) documented chronic absenteeism among public health workers, and Das et al. (2008) showed that provider effort and diagnostic accuracy were substantially lower in public than private settings, helping to explain initial patient preferences. More recent evidence confirms the pattern: BBS (2019) estimates that 58% of rural Bangladeshi households rely primarily on informal providers, and work by Ahmed and Hossain (2007) and Gautham et al. (2014) documents the relational factors—familiarity, proximity, social trust, and informal credit arrangements—that sustain informal provider loyalty independently of clinical quality.

The literature on demand-side barriers to public healthcare use in low and middle income countries (LMICs) identifies three distinct frictions. First, information barriers: many households lack accurate knowledge of public facility locations, hours, fees, or provider qualifications (Kremer and Glennerster 2011). Second, non-price access frictions: even when prices are known, travel time, queue uncertainty, and the cognitive burden of navigating bureaucratic systems deter use (Dupas 2014, Miller and Mobarak 2015). Third, trust and uncertainty: because healthcare has credence-good characteristics—treatment quality cannot be verified even after consumption—households rely on social networks and prior experience to form beliefs about provider quality (Darby and Karni 1973, Emons 1997). These frictions interact: when informal providers are highly observable on nonclinical dimensions, uncertainty about public care persists even when households receive factually accurate information.

Experimental and quasi-experimental studies of demand-side interventions in LMIC health settings have returned mixed results. Information campaigns improve knowledge but typically fail to change behavior (Oster and Thornton 2012, Banerjee et al. 2021). Financial subsidies can increase uptake but do not address underlying trust barriers (Dupas 2014). The present study provides direct evidence by comparing information, friction reduction, and assisted experience in a unified experimental design (Banerjee et al. 2004, Rahman et al. 2013, Das et al. 2016).

The results show that information alone is insufficient. These findings are consistent with awareness and behavior being decoupled because factors other than information, such as trust, familiarity, and prior experience, are the relevant constraints in this setting. While providing information substantially increased awareness by 33 percentage points (95% CI 25–41 pp; *P* < 0.001), it did not change actual behavior. In contrast, a single experience of obtaining formal medical advice had large effects within the 2-month follow-up window: households in the experience arm were 18 percentage points (95% CI 14–22 pp; *P* < 0.001) more likely to complete a public visit, and those induced to use public care reduced total health spending by 601 taka (USD 5.46 at 2023 exchange rate; 95% CI 373–829 taka; *P* < 0.001), driven primarily by lower medicine expenditures.

The findings are consistent with healthcare functioning as both a credence good and an experience good. A single successful interaction with a qualified provider can update beliefs, reduce uncertainty, and break habitual provider choice (Dupas 2014).

The paper makes three contributions. First, the findings suggest information campaigns may be insufficient when households place high value on familiarity and prior experience with existing informal providers. Second, one-time experimentation can generate short-run behavioral change within the follow-up window. Third, access to formal medical advice disciplines informal medicine markets and reduces unnecessary drug consumption.

## Materials and methods

### Study setting

Rural Bangladesh provides an ideal setting. Approximately 58% of rural households rely on local informal providers as their primary source of medical care (BBS 2019). Medicine purchases account for ∼60% of rural health expenditures (BBS 2019). Healthcare in Bangladesh has a credence goods problem: patients cannot verify treatment appropriateness even after consumption (Emons 1997), while patients observe functional attributes of care and often report high satisfaction with informal providers (Banerjee et al. 2004), creating habitual loyalty (Oliver 1999).

### Study design and participants

I conducted a cluster-randomized controlled trial across 600 paras across 29 upazilas (sub-districts) spanning all eight administrative divisions of Bangladesh. Randomization was stratified by administrative units (Bruhn and McKenzie 2009).

To maintain equal analytic sample sizes across treatment arms, one randomly selected household was excluded from the main treatment-effect analysis in every para. In treated paras (T1, T2, and T3), this household served as the spillover holdout: it was left untreated and was used exclusively to estimate within-para spillover effects. In control paras, one randomly selected household was excluded solely for sample balance and does not enter any analysis. The para is the unit of randomization; each para was assigned in its entirety to exactly one arm (control, T1, T2, or T3). Treatment is not randomized within paras. Of 600 paras initially selected across 29 upazilas, 580 entered the final study sample; within each para, five households were sampled at baseline, giving a full field sample of 2900 households. This procedure yields an analytic sample of 2320 households (580 per arm: 580 paras × 4 households per para). The main treatment-effect estimates use only the four included households per para across all arms; holdout and balance-excluded households are set aside entirely. A CONSORT participant flow diagram tracing all steps from 2900 sampled to 2320 analytic to 2261 endline households is provided as Figure A1 in the Appendix.

Between-para spillovers were tested by comparing outcomes in control paras within the same sub-district as treated paras against those in sub-districts with no treated paras. No significant between-para spillover effects were detected (all *P* > 0.15), consistent with the rural geography of the study, where provider relationships and social networks are predominantly local.

Inclusion criteria are as follows: households (i) located within a selected para, (ii) owning at least one functioning mobile phone, (iii) having at least one member serving as the primary healthcare decision-maker, (iv) having at least one child under 5 years of age, and (v) having sought care from a private or informal provider in the 3 months prior to baseline. Exclusion criteria are as follows: households (i) where the decision-maker was unavailable at both attempted visits, (ii) that had relocated, or (iii) already enrolled in a government health insurance or conditional cash transfer (CCT) program.

Baseline data were collected July 2022–January 2023. Intervention delivery took place June–August 2023. Enumerators completed 5-day residential training. Field supervisors conducted unannounced spot-checks on at least 5% of visits. Phone backchecks were conducted for 10%–12% of surveys; interviews with inconsistencies on more than two key items were re-administered. No systematic protocol deviation was identified.

### Interventions

**Treatment 1—Information:** Standardized information on fees, qualifications, and wait times delivered verbally with a printed flyer.

x**Treatment 2—Friction reduction:** Treatment 1 plus facility map, clinic hours, and where feasible, queue tokens or referral slips.

Queue tokens and referral slips were provided uniformly to all Treatment 2 households regardless of baseline proximity to public facilities. For households already proximate to a facility, these instruments may have functioned as commitment devices or social authorization cues rather than pure friction reducers, partially overlapping with the trust channel that Treatment 3 is intended to test. Heterogeneous Treatment 2 effects by baseline access constraints were not prespecified. This limits clean mechanism separation between Treatment 2 and Treatment 3; see the Limitations section.

**Treatment 3—Experience encouragement:** Treatment 2 plus a priority token, guided referral, or accompaniment to obtain a one-time assisted public visit.

### Outcomes and follow-up

Awareness outcomes (four binary measures, no recall period): (i) “Public healthcare exists”—unprompted awareness of at least one free/subsidized public service; (ii)”‘Can name a public facility”; (iii) “Aware of access point”; (iv) “Knows where to access care”.

Adoption outcomes (2-month recall): (i) “Attempted public visit”; (ii) “Completed public visit”; (iii) “Number of public visits”; (iv) “Switched from informal provider”, conditional on an illness episode occurring.

Health expenditures (2-month recall, taka/month): total expenditure, medicine expenditure, consultation fees, and diagnostic test expenditure.

Endline surveys were conducted in September–October 2023. Phased baseline data collection (July 2022–January 2023) means some clusters were surveyed in different illness seasons. Seasonal variation in illness type and severity is a known driver of provider choice in Bangladesh and represents a residual threat to internal validity. Season-of-baseline-interview controls are included in Table A3 robustness checks. Attrition was 2.54%, with no significant differences across arms (Table A1).

### Statistical analysis

Intent to treat (ITT) effects were estimated by ordinary least squares (OLS) with upazila fixed effects, baseline outcome controls, and imbalanced covariates. Standard errors were clustered at the para level. To estimate the causal effect of completing an in-person public visit, I used two-stage least squares (2SLS) on the full analytic sample (*N* = 2261 at endline). The endogenous variable is a binary indicator equal to one if the household completed at least one in-person public facility visit during the 2-month follow-up period. The instrument is assignment to Treatment 3. The first-stage coefficient on T3 assignment is 0.305 (SE = 0.044; *P* < 0.001); the first-stage F-statistic is 47.3, which substantially exceeds the conventional weak-instrument threshold of 10 (Stock and Yogo 2005). Missing data were minimal (<2%); analyses used complete-cases. Results are robust to winsorizing expenditure outcomes at the 90th and 95th percentiles (Table A3, columns 1–4) and to excluding baseline public-care satisfaction from the covariate set entirely rather than adjusting for it linearly (Table A3, column 5); the T3 estimates on completed visits and total expenditure are not materially affected by either alternative. Ethical approval was granted by the Institutional Review Board of the Ministry of Health, Bangladesh (Approval No.: BGD-IDB-1071130). All procedures conformed to the principles of the Declaration of Helsinki. Written informed consent was obtained from all participating households prior to data collection. This trial has been retrospectively registered with the AEA RCT Registry (AEARCTR-0019101). Registration occurred after data collection was complete; all main outcomes and specifications were defined prior to data collection, but a formal registry entry was not submitted at that time.

## Results

### Baseline characteristics and balance

At baseline, 1276 (55%) of 2320 households relied primarily on informal providers, and 2042 (88%) consulted informal sources for health information. Fewer than 116 (5%) were aware of free public healthcare services. Table 1 presents provider characteristics. Balance tests are in Table A2; four variables showed imbalance at the 10% level or better (respondent is household head, respondent education, household head education, and baseline public-care satisfaction) and are included as controls in all specifications (Table 1).

**Table 1:** Baseline characteristics of healthcare providers.

	Informal providers	Formal providers
Number of visits (*N*)^a^	2312	845
Share of visits (%)^a^	73.2	26.8
Mean consultation fee (Taka)^a^	40.6 (64.2)	195.1 (203.1)
Mean travel time (minutes)^a^	21.4 (23.1)	60.2 (31.3)
Mean travel cost (Taka)^a^	19.5 (36.7)	64.8 (113.4)
Mean satisfaction score (1–7)^b^	5.92 (0.62)	6.06 (0.65)

Notes: Informal providers include medicine sellers and unlicensed rural practitioners; formal providers include licensed public and private practitioners. Satisfaction measured on a seven-point Likert scale. Standard deviations in parentheses.

^a^Visit-level figures (based on all illness episodes reported at baseline).

^b^Household-level figure (mean across households).

### Effects on awareness

Information provision increased awareness by 33 pp (95% CI 25–41 pp; *P* < 0.001), ability to name a facility by 38 pp, and knowledge of where to access care by 37 pp. Friction reduction added no significant incremental awareness effects. Experience encouragement added 6–7 pp on narrower measures (*P* = 0.021–0.042). Holdout households showed higher awareness than pure controls, consistent with local information diffusion (Table 2).

**Table 2:** Effects on awareness of public healthcare options (ITT estimates).

	(1) Public healthcare exists	(2) Can name a public facility	(3) Aware of access point	(4) Knows where to access care
**Panel A: Intent-to-treat estimates (OLS)**				
Information provision (T1 vs control)	0.33 (0.25–0.41) *P < 0.001*	0.38 (0.30–0.46) *P < 0.001*	0.32 (0.24–0.40) *P < 0.001*	0.37 (0.29–0.45) *P < 0.001*
Friction reduction (incremental, T2 vs T1)	−0.03 (−0.09–0.03) *P* = *0.31*	−0.04 (−0.10–0.02) *P* = *0.19*	−0.02 (−0.08–0.04) *P* = *0.52*	0.01 (−0.05–0.07) *P* = *0.74*
Experience encouragement (incremental, T3 vs T2)	0.04 (−0.02–0.10) *P* = *0.18*	0.06 (0.00–0.12) *P* = *0.042*	0.06 (0.00–0.12) *P* = *0.038*	0.07 (0.01–0.13) *P* = *0.021*
Observations	2261	2261	2261	2261
Control mean	0.43	0.34	0.42	0.26

Notes: Outcome variables are binary indicators of awareness. ITT estimates by OLS. All specifications include upazila fixed effects, baseline outcome, and imbalanced covariates. Standard errors clustered at para level. 95% confidence intervals in parentheses.

### Effects on adoption

Information and friction reduction had no significant effect on completed public visits. Experience encouragement produced large effects: households in T3 were 25 percentage points more likely to attempt a public visit (95% CI 19–31 pp over control; *P* < 0.001) and 18 percentage points more likely to complete one (95% CI 14–22 pp; *P* < 0.001). Among control households that completed a public visit (9% of controls), only 9% used formal care again within 2 months, compared with 31% of T3 completers. No behavioral spillovers to holdout households were detected (all *P* > 0.20) (Table 3).

**Table 3:** Effects on adoption of public healthcare.

	(1) Attempted public visit	(2) Completed public visit	(3) No. of public visits	(4) Switched from informal provider
**Panel A: Intent-to-treat estimates (OLS)**				
Information provision	0.02 (−0.02–0.06) *P* = *0.35*	0.01 (−0.03–0.05) *P* = *0.60*	0.03 (−0.03–0.09) *P* = *0.33*	0.01 (−0.03–0.05) *P* = *0.62*
Friction reduction (incremental)	0.04 (0.00–0.08) *P* = *0.048*	0.03 (−0.01–0.07) *P* = *0.13*	0.06 (0.00–0.12) *P* = *0.042*	0.02 (−0.02–0.06) *P* = *0.31*
Experience encouragement (incremental)	0.25 (0.19–0.31) *P < 0.001*	0.18 (0.14–0.22) *P < 0.001*	0.31 (0.23–0.39) *P < 0.001*	0.14 (0.10–0.18) *P < 0.001*
**Panel B: Local average treatment effects (2SLS)**				
Public healthcare use	—	0.58 (0.42–0.74) *P < 0.001*	1.02 (0.76–1.28) *P < 0.001*	0.46 (0.32–0.60) *P < 0.001*
Observations	2261	2261	2261	2261
Control mean	0.12	0.09	0.14 visits	0.08

Notes: Panel A reports ITT estimates by OLS. Panel B reports LATE by 2SLS. The endogenous variable is a binary indicator equal to one if the household completed at least one in-person public facility visit during the 2-month follow-up period; the instrument is assignment to Treatment 3 (first-stage coefficient: 0.305, SE = 0.044; first-stage F = 47.3). All specifications include upazila fixed effects, baseline outcome, and imbalanced covariates. Standard errors clustered at para level.

### Effects on health expenditures

To contextualize, the control group mean total monthly health expenditure was 1893 taka (∼USD 17.22 at 2023 exchange rate), representing 12.1% of estimated monthly household income—above the 10% catastrophic expenditure threshold. Medicine expenditure (1142 taka; USD 10.38) accounted for 60% of total spending, consistent with national estimates for rural Bangladesh.

Information and friction reduction had no significant expenditure effects. Experience encouragement reduced total health expenditures by 183.4 taka (95% CI 83–284 taka; *P* < 0.001) and medicine expenditures by 142.6 taka (95% CI 73–212 taka; *P* < 0.001). Local average treatment effect (LATE) estimates (see Table 4, panel B and Table 3, panel B for full specification) indicate that households induced to use public care reduced total spending by 601.2 taka (USD 5.46 at 2023 exchange rate; 32%; 95% CI 373–829 taka; *P* < 0.001) and medicine expenditure by 467.8 taka (USD 4.25 at 2023 exchange rate; 41%; 95% CI 293–643 taka; *P* < 0.001). The control group mean health expenditure represents 12.1% of monthly household income, above the 10% catastrophic threshold; the estimated LATE reduction of 601 taka would lower this burden by ∼3.8 percentage points, a welfare improvement that is economically meaningful for rural poor households. The mechanism is substitution away from overprescribing informal providers (Table 4).

**Table 4:** Effects on health expenditures.

	(1) Total health expend. (Taka)	(2) Medicine expend. (Taka)	(3) Consultation fees (Taka)	(4) Diagnostic tests (Taka)
**Panel A: Intent-to-treat estimates (OLS)**				
Information provision	−28.4 (−111–54) *P* = *0.50*	−15.3 (−74–43) *P* = *0.61*	−3.2 (−19–13) *P* = *0.70*	−4.6 (−23–14) *P* = *0.62*
Friction reduction (incremental)	−61.2 (−148–26) *P* = *0.17*	−38.7 (−100–22) *P* = *0.21*	−5.8 (−22–11) *P* = *0.49*	−6.1 (−25–13) *P* = *0.54*
Experience encouragement (incremental)	−183.4 (−284–−83) *P < 0.001*	−142.6 (−212–−73) *P < 0.001*	−18.3 (−36–−1) *P* = *0.042*	−12.8 (−33–7) *P* = *0.21*
**Panel B: Local average treatment effects (2SLS)**				
Public healthcare use	−601.2 (−829–−373) *P < 0.001*	−467.8 (−643–−293) *P < 0.001*	−59.9 (−111–−9) *P* = *0.021*	−42.0 (−100–16) *P* = *0.16*
First-stage F-statistic	47.3	47.3	47.3	47.3
Observations	2261	2261	2261	2261
Control mean (Taka)	1893	1142	134	287

Notes: All expenditure outcomes in Bangladeshi taka per month. Panel A: ITT by OLS. Panel B: LATE by 2SLS (endogenous variable: completed in-person public facility visit; instrument: T3 assignment; first-stage F = 47.3). All specifications include upazila fixed effects, baseline outcome, and imbalanced covariates. Standard errors clustered at para level. Results robust to winsorizing at 90th and 95th percentiles (Table A3).

## Discussion

This study provides causal evidence that facilitated direct experience with public care, the T3 increment over information and access support, is an effective lever for shifting healthcare demand in rural Bangladesh. The ITT estimates isolate the incremental effect of experience encouragement over and above information provision and friction reduction. The results speak to a fundamental distinction that has been underappreciated in the demand-side intervention literature: the difference between not knowing and not wanting. Information campaigns raised awareness substantially but produced no change in actual provider choice. This is not a failure of message delivery—33 percentage points more households knew that free public care existed at endline. The findings are inconsistent with information alone being the primary constraint in this setting. The results are consistent with households placing substantial value on familiarity, convenience, geographic proximity, social trust, and prior experience with existing informal providers, dimensions on which informal providers hold a strong advantage rather than lacking information about public alternatives.

### Mechanisms: credence goods, experience goods, and the role of direct experience

The theoretical framework that best accounts for the pattern of results treats informal healthcare as functioning simultaneously as a credence good and an experience good, and public healthcare as a differentiated experience good whose attributes are unknown to households that have never used it. As a credence good (Darby and Karni 1973, Emons 1997), healthcare quality cannot be verified by patients even after consumption: a household prescribed antibiotics for a viral illness may recover without ever knowing whether the treatment was necessary or appropriate. This inability to verify quality ex post gives informal providers substantial scope to systematically overprescribe without losing patient trust, and it means that externally provided information about public care quality does not resolve the uncertainty patients face. Knowing that a public doctor is qualified is different from knowing whether a public consultation will be good—and in the absence of direct experience, households rationally discount quality claims about an unfamiliar provider.

As an experience good (Nelson 1970), healthcare quality is partly observable through direct use: patients can form assessments of waiting time, provider responsiveness, perceived thoroughness, and whether they felt heard and treated respectfully. On these dimensions, informal providers hold a systematic advantage that has been built through repeated interactions over time. The high baseline satisfaction scores for informal providers (5.92 out of 7) documented in Table 1 are not noise: they reflect genuine experiential assessments of providers who are embedded in the community, speak the same dialect, remember the household’s medical history, and are available without appointment. Public providers, by contrast, are evaluated against a prior that is shaped by decades of documented underperformance: absenteeism, understaffing, brusque interactions, and medicine stockouts (Chaudhury et al. 2006, Das et al. 2008). This prior cannot be updated by information alone; it requires direct interaction that contradicts it.

The experience arm works precisely because it substitutes direct evidence for the prior. A single successful consultation—one that is accessible, respectful, and perceived as effective—updates the household’s beliefs about public care quality across the dimensions that matter to them as an experience good. The pattern of repeat public care use is consistent with this belief-updating mechanism: 31% of Treatment 3 completers used public care again within 2 months, compared with 9% of control completers. This comparison is descriptive: it contrasts different populations within the same follow-up window and does not constitute causal evidence of persistence, but it is directionally consistent with belief updating having occurred. The mechanism is reinforced by the expenditure findings. The 41% reduction in medicine expenditure among induced public care users (LATE estimate: 467.8 taka; USD 4.25) reflects substitution away from informal providers who face revenue incentives to overprescribe. When a qualified public physician replaces an informal drug seller as the primary care contact, the scope for demand induction contracts sharply, consistent with Currie et al. (2011) on the role of patient knowledge in limiting antibiotic overuse.

### The Bangladesh context: why these mechanisms operate with particular force

The magnitude of the effects—an 18 percentage point increase in completed public visits and a 32% reduction in total health spending—reflects features of the Bangladesh rural health system that amplify the mechanisms described above. Three contextual factors deserve particular emphasis.

First, the depth of informal provider entrenchment. At baseline, 55% of sample households relied primarily on informal providers, and 88% used informal sources for health information. This is not a marginal phenomenon: informal providers are the primary healthcare institution for the majority of rural households. Their dominance reflects not just cost advantages—consultation fees of 40.6 taka versus 195.1 taka for formal providers—but also structural embeddedness. Village-level drug sellers and unlicensed practitioners (known locally as “palli chikitshak”) operate on informal credit, are available at all hours, and serve as the first point of contact for virtually all acute illness episodes. In this context, the default to informal care is not a considered choice made at each illness episode; it is a habit formed over years of satisfactory low-cost interaction. Breaking such habits requires not persuasion but experience (Oliver 1999).

Second, the historically poor reputation of public facilities. Rural public health facilities in Bangladesh have faced persistent staffing crises: Chaudhury et al. (2006) documented absenteeism rates among public health workers that averaged 35% across South Asia, and qualitative evidence from Bangladesh consistently reports that rural households associate public facilities with long waiting times, absent doctors, empty medicine cabinets, and impersonal treatment (Ahmed and Hossain 2007, Mia et al. 2020). These prior beliefs are not irrational given past experience, and they persist even as public facility quality has improved in some dimensions through recent reforms including the community health clinic program and mobile health services. The information-to-behavior gap observed in Treatment 1 is fully consistent with a household that has updated its factual knowledge of public care availability but not its experiential prior about public care quality: households knew more but remained satisfied with informal care. The experience arm is effective precisely because it creates a new data point that contradicts that prior directly.

Third, the antimicrobial resistance dimension. Medicine expenditure accounts for 60% of total health spending in the control group (1142 taka out of 1893 taka per month), and the majority of this spending flows to informal providers who have strong financial incentives to recommend medicines—including antibiotics—at rates that far exceed clinical need (Ahmed and Hossain 2007). The 41% reduction in medicine expenditure among induced public care users is therefore not just an economic finding; it is a public health finding with direct implications for antimicrobial resistance. Bangladesh has among the highest rates of inappropriate antibiotic use in low-income countries, and the informal provider sector is a primary driver (WHO and World Bank 2017). Interventions that shift households from informal to public care even temporarily reduce antibiotic exposure and may contribute to slowing resistance development at the population level.

### Policy implications

The findings carry three distinct policy implications for the design of rural health programs in Bangladesh and comparable LMIC settings.

First, information campaigns may not be a cost-effective instrument for shifting provider choice when the findings suggest that factors beyond information, such as trust, familiarity, and prior experience, are the relevant constraints. The Government of Bangladesh and development partners have invested substantially in health communication campaigns—through community health workers, radio, and digital platforms—aimed at increasing awareness of free public services. The present results show that such campaigns can achieve their proximate goal (awareness increases sharply) while failing to achieve their ultimate goal (utilization does not change). This does not mean information campaigns are without value: there may be subpopulations with genuinely low awareness for whom information is a binding constraint. But for the majority of rural households already embedded in informal provider networks, the findings suggest information is unlikely to be the primary lever in such settings, and resources spent on awareness campaigns alone may not translate into utilization gains.

Second, low-cost experience-facilitation components should be incorporated into existing health programs as standard practice. The marginal cost of adding a priority token, a guided referral slip, or an accompanied first facility referral to an existing information or health worker visit is small relative to the behavioral returns documented here. Bangladesh’s existing primary care infrastructure provides platforms for delivering experience-facilitation at scale. The Government of Bangladesh operates community health clinics supported by Community Health Care Providers, Health Assistants, and Family Welfare Assistants under the Ministry of Health and Family Welfare. In parallel, BRAC (formerly Bangladesh Rural Advancement Committee) operates its own extensive community health worker network comprising Shasthya Shebika and Shasthya Kormi, which reaches millions of households in rural Bangladesh independently of the government system. These two systems are complementary in some areas and overlapping in others. Either platform, where appropriate given local program coverage, could deliver experience-facilitation components such as guided facility referrals and accompanied first visits at modest marginal cost, without requiring new infrastructure. It should be noted that BRAC’s network is available only in areas where BRAC operates and that the appropriate delivery channel will depend on local health system configuration.

Third, the expenditure effects point to a self-reinforcing dynamic that amplifies the long-run value of experience-facilitation. The 32% reduction in total health spending (USD 5.46 at 2023 exchange rate) observed in the 2-month follow-up period represents a meaningful reduction relative to baseline spending that itself exceeded the 10% catastrophic expenditure threshold. For a rural household spending 1893 taka per month on health, this reduction frees resources for food, education, and other welfare-enhancing expenditures. More importantly, lower health spending reduces the financial burden of illness, which may lower barriers to care-seeking in subsequent episodes, creating a positive feedback loop: households that experience affordable, adequate public care are more likely to return, less likely to accumulate health debt, and less exposed to the overtreatment cycle that drives informal provider spending. This compounding dynamic is not visible in a 2-month follow-up and represents a key motivation for longer-term evaluation.

### Scaling trade-offs and boundary conditions

The policy implications described above must be qualified by an honest account of the conditions under which experience-facilitation is likely to work and the constraints on scaling.

The most important scaling constraint is supply-side capacity. The experience arm in this study required enumerator accompaniment, priority access arrangements with facility staff, and in some cases physical co-presence during the consultation. These components are feasible in a research setting but difficult to replicate at scale without dedicated community health worker capacity. If experience-facilitation is implemented through existing health worker networks without additional resources, the quality of the first experience may be lower than in the experimental setting, potentially reducing the belief-updating effect. Worse, if facilitated visits lead to congestion at facilities that are already understaffed, the quality of the experience may deteriorate precisely as demand grows, eroding the trust that the intervention was designed to build (see also the supply-side congestion limitation discussed below). Scaling must therefore be accompanied by investments in public facility staffing and service quality, not just demand-side facilitation.

A second boundary condition concerns the durability of the effect. The 2-month follow-up window documents a significant shift in provider choice and spending, but it cannot establish whether the effect lasts 6 months or 2 years. McKenzie (2012) has shown that short-run effects of demand-side interventions in development field experiments can fade as novelty effects dissipate and as social network influences reassert themselves. In the present context, the durability of the effect is likely to depend on the quality of the second and third public care interactions, which are outside the scope of the intervention. A household whose first facilitated experience is positive but whose second self-initiated visit is poor—because of a different doctor, a longer wait, or a stockout—may revert to their informal provider default. The experience-facilitation mechanism is therefore most powerful when public care quality is consistently good, which reinforces the complementarity between demand-side interventions and supply-side quality investments.

A third boundary condition concerns heterogeneity across household types. The sample is restricted to households with at least one child under five, who face an acute and regularly recurring health risk that generates frequent illness episodes and high provider contact. Such households have a strong revealed preference for accessible, responsive care, and may be particularly receptive to an experience that demonstrates public care can deliver this. Households without young children, elderly populations managing chronic conditions, and households in more remote areas with genuinely poor physical access to public facilities may respond differently. The experience-facilitation mechanism requires that a reasonably good experience is feasible—which depends on geographic proximity and facility quality—and that the household has a recurring health need that gives the shift in provider preference a chance to manifest in changed behavior. Policymakers should not assume that a single experience-facilitation model will work uniformly across all population segments, and targeting of experience-facilitation components toward high-contact, high-need households is likely to generate the greatest returns per unit cost.

### Limitations

Several limitations deserve explicit acknowledgement. First, mechanism identification in Treatment 2: queue tokens and referral slips were provided uniformly regardless of baseline proximity. For households already near a public facility, these may have functioned as commitment devices or trust signals rather than pure friction reducers, limiting clean mechanism separation between Treatments 2 and 3.

Second, social desirability and Hawthorne bias: treated households face social desirability pressure to report public care use consistent with the intervention message, independently of actual behavior. This differential reporting bias may inflate utilization estimates across arms and cannot be ruled out in the absence of administrative validation of reported visits.

Third, supply-side congestion: the study does not address whether public facilities can absorb induced demand without quality deterioration. If congestion erodes the interpersonal quality of care that generated the initial trust shift, the experience effect may be self-limiting at scale—a critical qualification for the policy recommendations, particularly given documented staffing constraints in Bangladesh’s public sector (Chaudhury et al. 2006).

Fourth, gender and household decision-making: the eligibility criterion does not ensure the intervention reached the primary care decision-maker. Gender and household head education are well-documented predictors of care-seeking for children under five in Bangladesh, and if the intervention reached a non-primary decision-maker, effect estimates may be attenuated in ways not apparent from the study design.

Fifth, seasonality: phased baseline data collection means some clusters were surveyed in different illness seasons. Seasonal variation in illness type and severity is a known driver of provider choice in Bangladesh and represents a residual threat to internal validity, though robustness checks with season-of-baseline-interview controls are reported in Table A3.

Outcome measures rely on self-reported data. The follow-up period spans only 2 months; longer-term follow-up is needed to establish durability of effects (McKenzie 2012). The sample is restricted to households with at least one child under five, limiting generalizability to other demographic groups. The study does not directly measure clinical quality or health outcomes.

## Conclusion

Modest, low-cost interventions that facilitate a single positive experience with public healthcare can produce meaningful changes in provider choice and health expenditures in rural Bangladesh. Information campaigns raise awareness but are insufficient when households are satisfied with informal care—the relevant barrier is not ignorance but experience. Experience-based interventions update beliefs about public care quality, reduce reliance on overprescribing informal providers, and lower out-of-pocket health spending by a magnitude that is economically significant relative to rural household incomes. These findings have direct implications for health systems policy in LMICs where informal providers dominate primary care and antimicrobial resistance is a growing concern.

## Supplementary Material

czag109_Supplementary_Data

## Figures and Tables

**Figure A1.**
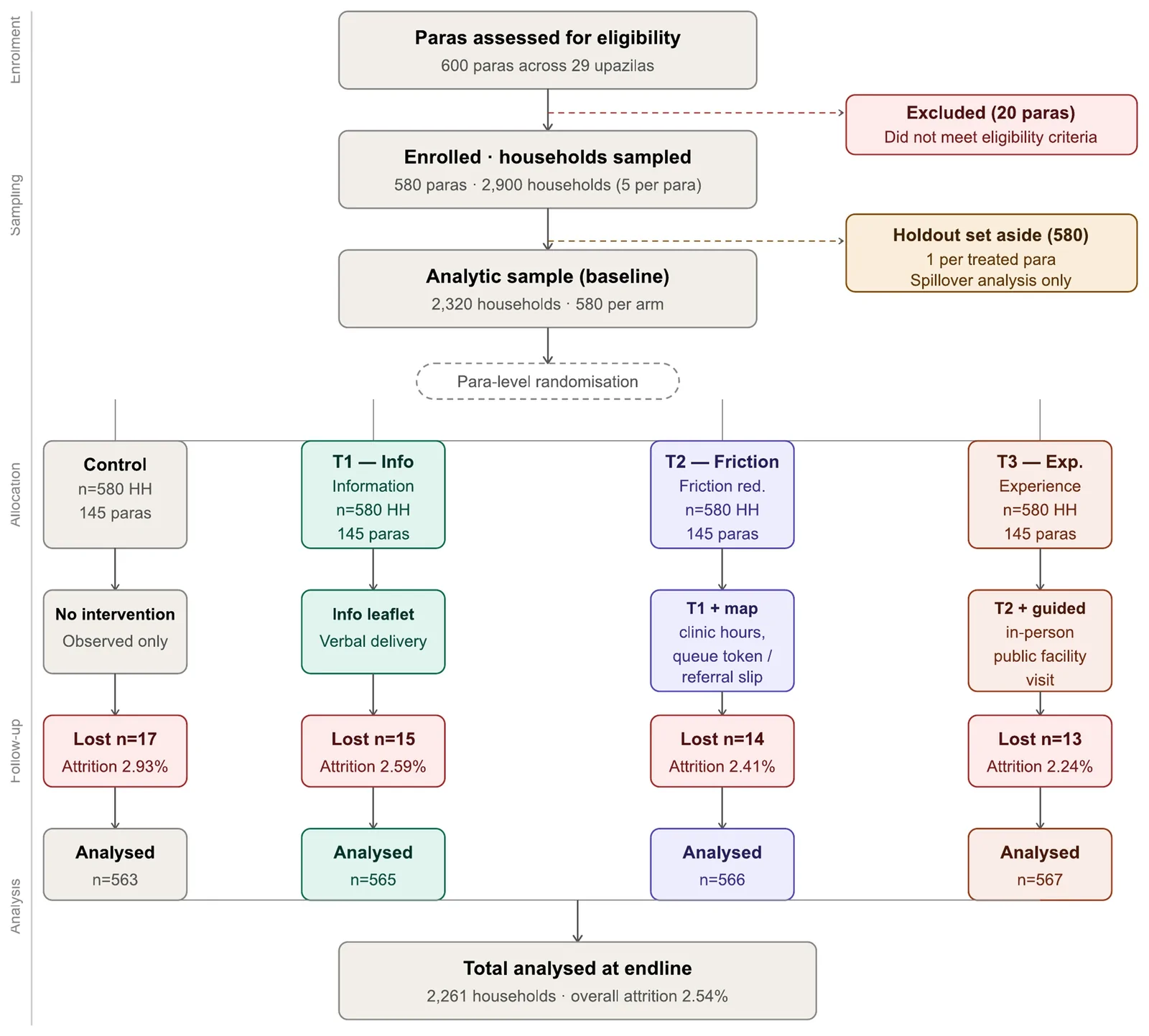
CONSORT participant flow diagram. Flow of participants from eligibility assessment through randomization, intervention, follow-up, and analysis. Of 600 paras assessed for eligibility, 580 were enrolled and randomized across four arms (control, T1, T2, T3), with 580 households per arm in the analytic sample (2320 total). Endline data were available for 2261 households (attrition 2.54%). Holdout households (*n* = 580, one per treated para) and balance-excluded households (*n* = 580, one per control para) are shown separately and were not included in the main analysis.

## Data Availability

The data supporting this study contain sensitive participant information and are not publicly available. De-identified data may be made available from the corresponding author upon reasonable request and subject to approval by the relevant IRB and data use agreements.

## Trial registration

This trial has been retrospectively registered with the AEA RCT Registry (AEARCTR-0019101). Registration occurred after data collection was complete; all main outcomes and specifications were defined prior to data collection, but a formal registry entry was not submitted at that time.

## Reflexivity statement

This paper is the work of a single male author at the assistant professor level. The author is Bangladeshi by origin and currently based in the USA, bringing direct familiarity with the country context alongside training in development and health economics. The research was conducted entirely in rural Bangladesh, and the author’s background in the local institutional environment informed both the study design and the interpretation of findings.

## Appendix 1

**Table A1.** Attrition by treatment arm

	Control	T1 (information)	T2 (friction reduction)	T3 (experience)
Baseline households (analytic sample)	580	580	580	580
Endline households	563	565	566	567
Attrition rate (%)	2.93	2.59	2.41	2.24
Difference from control	—	−0.34 (−1.26, 0.58) *P* = 0.47	−0.52 (−1.42, 0.38) *P* = 0.26	−0.69 (−1.57, 0.19) *P* = 0.12

Notes: Analytic sample excludes one randomly selected household per para (580 total): in treated paras, this household is the spillover holdout; in control paras, one household was excluded for sample balance. Baseline households shown are those in the analytic sample (*N* = 2320 total; 580 per arm). Differences relative to the control group with 95% confidence intervals and *P*-values. Joint *P*-value tests equality of attrition rates across all arms (joint *P* = 0.61). No statistically significant differences in attrition detected.

**Table A2.** Baseline balance—descriptive statistics and orthogonality tests

Variable	Control mean (SD)	T1−Control (SE)	T2−Control (SE)	T3−Control (SE)
Household members	5.29 (1.83)	0.00 (0.11)	−0.04 (0.11)	0.06 (0.11)
Respondent is household head^a^	0.43 (0.50)	−0.03 (0.03)	−0.01 (0.03)	0.05^c^ (0.03)
Respondent education (years)^a^	6.86 (4.23)	0.68^b^ (0.25)	0.24 (0.25)	0.16 (0.24)
Household head education (years)^a^	5.95 (4.44)	0.67^b^ (0.27)	0.26 (0.27)	0.49^c^ (0.25)
Main income: employment	0.36 (0.48)	−0.02 (0.03)	−0.03 (0.03)	0.00 (0.03)
Monthly household income (Taka)	15,657 (9,584)	117.8 (652.5)	156.4 (648.2)	961.2 (621.3)
Monthly health expenditure (Taka)	1,893 (3,523)	321.7 (218.4)	156.1 (218.0)	49.2 (209.4)
Baseline public care satisfaction^a^	5.07 (1.82)	0.08 (0.10)	0.13 (0.10)	0.21^b^ (0.10)
Aware of public HC at baseline	0.43 (0.49)	−0.01 (0.03)	0.01 (0.03)	0.02 (0.03)

Notes: Control group means with standard deviations in parentheses. T1–T3 columns show differences from the control mean with standard errors in parentheses.

^a^Four variables showing imbalance at the 10% level or better, included as controls in all main specifications.

^b^ *P* < 0.05.

^c^ *P* < 0.10.

**Table A3.** Robustness checks—key estimates under alternative specifications

	(1) Main specification	(2) No baseline controls	(3) HC2 robust SEs	(4) Wild bootstrap *P*-value	(5) Excl. baseline satisfaction
**Panel A: Awareness**—**information arm (T1)**					
Change in probability (95% CI)	0.33 (0.25–0.41)	0.35 (0.27–0.43)	0.33 (0.25–0.41)	—	0.33 (0.25–0.41)
*P*-value	*P* < 0.001	*P* < 0.001	*P* < 0.001	*P* = 0.001	*P* < 0.001
**Panel B: Awareness**—**experience arm (T3 incremental)**					
Change in probability (95% CI)	0.07 (0.01–0.13)	0.07 (0.01–0.13)	0.07 (0.01–0.13)	—	0.07 (0.01–0.13)
*P*-value	*P* = 0.021	*P* = 0.021	*P* = 0.021	*P* = 0.032	*P* = 0.022
**Panel C: Completed public visits**—**experience arm**					
Change in probability (95% CI)	0.18 (0.14–0.22)	0.19 (0.15–0.23)	0.18 (0.14–0.22)	—	0.17 (0.13–0.21)
*P*-value	*P* < 0.001	*P* < 0.001	*P* < 0.001	*P* < 0.001	*P* < 0.001
**Panel D: Total health expenditure**—**experience arm**					
Change in expenditure (95% CI)	−183.4 (−284–−83)	−191.2 (−294–−88)	−183.4 (−288–−79)	—	−178.2 (−279–−78)
*P*-value	*P* < 0.001	*P* < 0.001	*P* < 0.001	*P* = 0.001	*P* < 0.001
Observations	2261	2261	2261	2261	2261

Notes: Column (1) main specification (upazila fixed effects, baseline outcome, four imbalanced covariates). Column (2) omits baseline controls. Column (3) uses HC2 robust standard errors. Column (4) reports wild bootstrap *P*-values (999 replications). Column (5) excludes baseline public-care satisfaction from the covariate set entirely rather than adjusting for it linearly; estimates for completed visits (panel C: 0.17 pp) and total expenditure (panel D: −178 taka) are not materially different from the main specification, confirming that results are not driven by differential baseline satisfaction in the T3 arm. Season-of-baseline-interview controls included in all columns. 95% CIs based on cluster-robust standard errors in columns 1–3.
