# Supplementary material for: Evidence on information, access frictions, and experience in public healthcare use in rural Bangladesh: a cluster-randomized field experiment
Source: Health Policy Plan. 2026 Sep 7;41(9):1580–90. doi: 10.1093/heapol/czag109 (PMC13678817; doi:10.1093/heapol/czag109)
Supplement: Supplementary file 1 [file czag109_supplementary_data.zip › CONSORT_Checklist_Filled.docx]

***CONSORT 2025 checklist of information to include when reporting a randomised trial***

| **Section / Topic** | **No** | **CONSORT 2025 checklist item description** | **Reported on page no.** |
| --- | --- | --- | --- |
| **Title and abstract** | | |  |
| Title and structured abstract | 1a | Identification as a randomised trial | Title page |
|  | 1b | Structured summary of the trial design, methods, results, and conclusions | Abstract |
| **Open science** | | |  |
| Trial registration | 2 | Name of trial registry, identifying number (with URL) and date of registration | Statistical analysis section |
| Protocol and statistical analysis plan | 3 | Where the trial protocol and statistical analysis plan can be accessed | Data Availability section; no SAP registered |
| Data sharing | 4 | Where and how the individual de-identified participant data (including data dictionary), statistical code and any other materials can be accessed | Data Availability section |
| Funding and conflicts of interest | 5a | Sources of funding and other support (e.g., supply of drugs), and role of funders in the design, conduct, analysis and reporting of the trial | Funding section |
|  | 5b | Financial and other conflicts of interest of the manuscript authors | Competing Interests section |
| **Introduction** | | |  |
| Background and rationale | 6 | Scientific background and rationale | Introduction |
| Objectives | 7 | Specific objectives related to benefits and harms | Introduction (final paragraph) |
| **Methods** | | |  |
| Patient and public involvement | 8 | Details of patient or public involvement in the design, conduct and reporting of the trial | NA - not applicable |
| Trial design | 9 | Description of trial design including type of trial (e.g., parallel group, crossover), allocation ratio, and framework (e.g., superiority, equivalence, non-inferiority, exploratory) | Study design and participants section |
| Changes to trial protocol | 10 | Important changes to the trial after it commenced including any outcomes or analyses that were not prespecified, with reason | Statistical analysis section; no changes to protocol |
| Trial setting | 11 | Settings (e.g., community, hospital) and locations (e.g., countries, sites) where the trial was conducted | Study setting section |
| Eligibility criteria | 12a | Eligibility criteria for participants | Study design and participants section (Inclusion/Exclusion criteria) |
|  | 12b | If applicable, eligibility criteria for sites and for individuals delivering the interventions (e.g., surgeons, physiotherapists) | NA - not applicable |
| Intervention and comparator | 13 | Intervention and comparator with sufficient details to allow replication. If relevant, where additional materials describing the intervention and comparator (e.g., intervention manual) can be accessed | Interventions section (Treatments 1-3) |
| Outcomes | 14 | Pre-specified primary and secondary outcomes, including the specific measurement variable (e.g., systolic blood pressure), analysis metric (e.g., change from baseline, final value, time to event), method of aggregation (e.g., median, proportion), and time point for each outcome | Outcomes and follow-up section |
| Harms | 15 | How harms were defined and assessed (e.g., systematically, non-systematically) | NA - harms not assessed in this demand-side intervention |
| Sample size | 16a | How sample size was determined, including all assumptions supporting the sample size calculation | NA - sample size determined by available clusters |
|  | 16b | Explanation of any interim analyses and stopping guidelines | NA - no interim analyses |
| Randomisation: |  |  |  |
| Sequence generation | 17a | Who generated the random allocation sequence and the method used | Study design and participants section |
|  | 17b | Type of randomisation and details of any restriction (e.g., stratification, blocking and block size) | Study design and participants section (stratified by upazila) |
| Allocation concealment mechanism | 18 | Mechanism used to implement the random allocation sequence (e.g., central computer/telephone; sequentially numbered, opaque, sealed containers), describing any steps to conceal the sequence until interventions were assigned | Study design and participants section |
| Implementation | 19 | Whether the personnel who enrolled and those who assigned participants to the interventions had access to the random allocation sequence | Study design and participants section |
| Blinding | 20a | Who was blinded after assignment to interventions (e.g., participants, care providers, outcome assessors, data analysts) | NA - blinding not feasible in behavioural intervention |
|  | 20b | If blinded, how blinding was achieved and description of the similarity of interventions | NA - blinding not feasible in behavioural intervention |
| Statistical methods | 21a | Statistical methods used to compare groups for primary and secondary outcomes, including harms | Statistical analysis section |
|  | 21b | Definition of who is included in each analysis (e.g., all randomised participants), and in which group | Statistical analysis section |
|  | 21c | How missing data were handled in the analysis | Statistical analysis section (missing data <2%, complete case) |
|  | 21d | Methods for any additional analyses (e.g., subgroup and sensitivity analyses), distinguishing prespecified from post-hoc | Statistical analysis section; Table A3 |
| **Results** | | |  |
| Participant flow, including flow diagram | 22a | For each group, the numbers of participants who were randomly assigned, received intended intervention, and were analysed for the primary outcome | Figure A1 (Appendix); Tables 2-4 |
|  | 22b | For each group, losses and exclusions after randomisation, together with reasons | Figure A1 (Appendix); Table A1 |
| Recruitment | 23a | Dates defining the periods of recruitment and follow-up for outcomes of benefits and harms | Study design and participants section (July 2022-October 2023) |
|  | 23b | If relevant, why the trial ended or was stopped | NA - trial not stopped early |
| Intervention and comparator delivery | 24a | Intervention and comparator as they were actually administered (e.g., where appropriate, who delivered the intervention/comparator, how participants adhered, whether they were delivered as intended [fidelity]) | Interventions section; Study design and participants section |
|  | 24b | Concomitant care received during the trial for each group | NA - not assessed |
| Baseline data | 25 | A table showing baseline demographic and clinical characteristics for each group | Baseline characteristics and balance section; Table 1; Table A2 |
| Numbers analysed,  outcomes and estimation | 26 | For each primary and secondary outcome, by group:   - the number of participants included in the analysis - the number of participants with available data at the outcome time point - result for each group, and the estimated effect size and its precision (such as 95% confidence interval) - for binary outcomes, presentation of both absolute and relative effect size | Results section; Tables 2, 3, 4 |
| Harms | 27 | All harms or unintended events in each group | NA - harms not assessed |
| Ancillary analyses | 28 | Any other analyses performed, including subgroup and sensitivity analyses, distinguishing pre-specified from post-hoc | Statistical analysis section; Table A3 |
| **Discussion** | | |  |
| Interpretation | 29 | Interpretation consistent with results, balancing benefits and harms, and considering other relevant evidence | Discussion section |
| Limitations | 30 | Trial limitations, addressing sources of potential bias, imprecision, generalisability, and, if relevant, multiplicity of analyses | Limitations section |

*We strongly recommend reading this statement in conjunction with the CONSORT 2025 Explanation and Elaboration and/or the CONSORT 2025 Expanded Checklist for important clarifications on all the items. We also recommend reading relevant CONSORT extensions. See [www.consort-spirit.org](http://www.consort-spirit.org).

Citation: Hopewell S, Chan AW, Collins GS, Hróbjartsson A, Moher D, Schulz KF, et al. CONSORT 2025 Statement: updated guideline for reporting randomised trials. BMJ. 2025; 388:e081123. <https://dx.doi.org/10.1136/bmj-2024-081123>.

© 2025 Hopewell et al. This is an Open Access article distributed under the terms of the Creative Commons Attribution License (<https://creativecommons.org/licenses/by/4.0/>), which permits unrestricted use, distribution, and reproduction in any medium, provided the original work is properly cited.
