# Supplementary material for: Evidence on information, access frictions, and experience in public healthcare use in rural Bangladesh: a cluster-randomized field experiment
Source: Health Policy Plan. 2026 Sep 7;41(9):1580–90. doi: 10.1093/heapol/czag109 (PMC13678817; doi:10.1093/heapol/czag109)
Supplement: Supplementary file 1 [file czag109_supplementary_data.zip › Table1.docx]

**Table 1. Baseline Characteristics of Healthcare Providers**

|  | **Informal providers** | **Formal providers** |
| --- | --- | --- |
| Number of visits (N)ᵃ | 2,312 | 845 |
| Share of visits (%)ᵃ | 73.2 | 26.8 |
| Mean consultation fee (Taka)ᵃ | 40.6 (64.2) | 195.1 (203.1) |
| Mean travel time (minutes)ᵃ | 21.4 (23.1) | 60.2 (31.3) |
| Mean travel cost (Taka)ᵃ | 19.5 (36.7) | 64.8 (113.4) |
| Mean satisfaction score (1–7)ᵇ | 5.92 (0.62) | 6.06 (0.65) |

Notes: Superscript a = visit-level figures (based on all illness episodes reported at baseline); superscript b = household-level figure (mean across households). Informal providers include medicine sellers and unlicensed rural practitioners; formal providers include licensed public and private practitioners. Satisfaction measured on a seven-point Likert scale. Standard deviations in parentheses.
