# Supplementary material for: Evidence on information, access frictions, and experience in public healthcare use in rural Bangladesh: a cluster-randomized field experiment
Source: Health Policy Plan. 2026 Sep 7;41(9):1580–90. doi: 10.1093/heapol/czag109 (PMC13678817; doi:10.1093/heapol/czag109)
Supplement: Supplementary file 1 [file czag109_supplementary_data.zip › Table2.docx]

**Table 2. Effects on Awareness of Public Healthcare Options (ITT Estimates)**

|  | **(1) Public healthcare exists** | **(2) Can name a public facility** | **(3) Aware of access point** | **(4) Knows where to access care** |
| --- | --- | --- | --- | --- |
| **Panel A: Intent-to-treat estimates (OLS)** | | | | |
| Information provision (T1 vs control) | 0.33  (0.25–0.41)  *p<0.001* | 0.38  (0.30–0.46)  *p<0.001* | 0.32  (0.24–0.40)  *p<0.001* | 0.37  (0.29–0.45)  *p<0.001* |
| Friction reduction (incremental, T2 vs T1) | −0.03  (−0.09–0.03)  *p=0.31* | −0.04  (−0.10–0.02)  *p=0.19* | −0.02  (−0.08–0.04)  *p=0.52* | 0.01  (−0.05–0.07)  *p=0.74* |
| Experience encouragement (incremental, T3 vs T2) | 0.04  (−0.02–0.10)  *p=0.18* | 0.06  (0.00–0.12)  *p=0.042* | 0.06  (0.00–0.12)  *p=0.038* | 0.07  (0.01–0.13)  *p=0.021* |
| Observations | 2,261 | 2,261 | 2,261 | 2,261 |
| Control mean | 0.43 | 0.34 | 0.42 | 0.26 |

Notes: Outcome variables are binary indicators of awareness. ITT estimates by OLS. All specifications include upazila fixed effects, baseline outcome, and imbalanced covariates. Standard errors clustered at para level. 95% confidence intervals in parentheses.
