# Supplementary material for: Evidence on information, access frictions, and experience in public healthcare use in rural Bangladesh: a cluster-randomized field experiment
Source: Health Policy Plan. 2026 Sep 7;41(9):1580–90. doi: 10.1093/heapol/czag109 (PMC13678817; doi:10.1093/heapol/czag109)
Supplement: Supplementary file 1 [file czag109_supplementary_data.zip › Table3 (1).docx]

**Table 3. Effects on Adoption of Public Healthcare**

|  | **(1) Attempted public visit** | **(2) Completed public visit** | **(3) No. of public visits** | **(4) Switched from informal provider** |
| --- | --- | --- | --- | --- |
| **Panel A: Intent-to-treat estimates (OLS)** | | | | |
| Information provision | 0.02  (−0.02–0.06)  *p=0.35* | 0.01  (−0.03–0.05)  *p=0.60* | 0.03  (−0.03–0.09)  *p=0.33* | 0.01  (−0.03–0.05)  *p=0.62* |
| Friction reduction (incremental) | 0.04  (0.00–0.08)  *p=0.048* | 0.03  (−0.01–0.07)  *p=0.13* | 0.06  (0.00–0.12)  *p=0.042* | 0.02  (−0.02–0.06)  *p=0.31* |
| Experience encouragement (incremental) | 0.25  (0.19–0.31)  *p<0.001* | 0.18  (0.14–0.22)  *p<0.001* | 0.31  (0.23–0.39)  *p<0.001* | 0.14  (0.10–0.18)  *p<0.001* |
| **Panel B: Local average treatment effects (2SLS)** | | | | |
| Public healthcare use | — | 0.58  (0.42–0.74)  *p<0.001* | 1.02  (0.76–1.28)  *p<0.001* | 0.46  (0.32–0.60)  *p<0.001* |
| Observations | 2,261 | 2,261 | 2,261 | 2,261 |
| Control mean | 0.12 | 0.09 | 0.14 visits | 0.08 |

Notes: Panel A reports ITT estimates by OLS. Panel B reports LATE by 2SLS. The endogenous variable is a binary indicator equal to one if the household completed at least one in-person public facility visit during the two-month follow-up period; the instrument is assignment to Treatment 3 (first-stage coefficient: 0.305, SE=0.044; first-stage F=47.3). All specifications include upazila fixed effects, baseline outcome, and imbalanced covariates. Standard errors clustered at para level.
