# Supplementary material for: Evidence on information, access frictions, and experience in public healthcare use in rural Bangladesh: a cluster-randomized field experiment
Source: Health Policy Plan. 2026 Sep 7;41(9):1580–90. doi: 10.1093/heapol/czag109 (PMC13678817; doi:10.1093/heapol/czag109)
Supplement: Supplementary file 1 [file czag109_supplementary_data.zip › Table4 (1).docx]

**Table 4. Effects on Health Expenditures**

|  | **(1) Total health expend. (Taka)** | **(2) Medicine expend. (Taka)** | **(3) Consultation fees (Taka)** | **(4) Diagnostic tests (Taka)** |
| --- | --- | --- | --- | --- |
| **Panel A: Intent-to-treat estimates (OLS)** | | | | |
| Information provision | −28.4  (−111–54)  *p=0.50* | −15.3  (−74–43)  *p=0.61* | −3.2  (−19–13)  *p=0.70* | −4.6  (−23–14)  *p=0.62* |
| Friction reduction (incremental) | −61.2  (−148–26)  *p=0.17* | −38.7  (−100–22)  *p=0.21* | −5.8  (−22–11)  *p=0.49* | −6.1  (−25–13)  *p=0.54* |
| Experience encouragement (incremental) | −183.4  (−284–−83)  *p<0.001* | −142.6  (−212–−73)  *p<0.001* | −18.3  (−36–−1)  *p=0.042* | −12.8  (−33–7)  *p=0.21* |
| **Panel B: Local average treatment effects (2SLS)** | | | | |
| Public healthcare use | −601.2  (−829–−373)  *p<0.001* | −467.8  (−643–−293)  *p<0.001* | −59.9  (−111–−9)  *p=0.021* | −42.0  (−100–16)  *p=0.16* |
| First-stage F-statistic | 47.3 | 47.3 | 47.3 | 47.3 |
| Observations | 2,261 | 2,261 | 2,261 | 2,261 |
| Control mean (Taka) | 1,893 | 1,142 | 134 | 287 |

Notes: All expenditure outcomes in Bangladeshi taka per month. Panel A: ITT by OLS. Panel B: LATE by 2SLS (endogenous variable: completed in-person public facility visit; instrument: T3 assignment; first-stage F=47.3). All specifications include upazila fixed effects, baseline outcome, and imbalanced covariates. Standard errors clustered at para level. Results robust to winsorising at 90th and 95th percentiles (Table A3).
