# Supplementary material for: Evidence on information, access frictions, and experience in public healthcare use in rural Bangladesh: a cluster-randomized field experiment
Source: Health Policy Plan. 2026 Sep 7;41(9):1580–90. doi: 10.1093/heapol/czag109 (PMC13678817; doi:10.1093/heapol/czag109)
Supplement: Supplementary file 1 [file czag109_supplementary_data.zip › TableA1.docx]

**Table A1. Attrition by Treatment Arm**

|  | **Control** | **T1 (Information)** | **T2 (Friction reduction)** | **T3 (Experience)** |
| --- | --- | --- | --- | --- |
| Baseline households (analytic sample) | 580 | 580 | 580 | 580 |
| Endline households | 563 | 565 | 566 | 567 |
| Attrition rate (%) | 2.93 | 2.59 | 2.41 | 2.24 |
| Difference from control | — | −0.34 (−1.26, 0.58) p=0.47 | −0.52 (−1.42, 0.38) p=0.26 | −0.69 (−1.57, 0.19) p=0.12 |

Notes: Analytic sample excludes one randomly selected household per para (580 total): in treated paras, this household is the spillover holdout; in control paras, one household was excluded for sample balance. Baseline households shown are those in the analytic sample (N=2,320 total; 580 per arm). Differences relative to control group with 95% confidence intervals and p-values. Joint p-value tests equality of attrition rates across all arms (joint p=0.61). No statistically significant differences in attrition detected.
