# Supplementary material for: Evidence on information, access frictions, and experience in public healthcare use in rural Bangladesh: a cluster-randomized field experiment
Source: Health Policy Plan. 2026 Sep 7;41(9):1580–90. doi: 10.1093/heapol/czag109 (PMC13678817; doi:10.1093/heapol/czag109)
Supplement: Supplementary file 1 [file czag109_supplementary_data.zip › TableA2.docx]

**Table A2. Baseline Balance — Descriptive Statistics and Orthogonality Tests**

| **Variable** | **Control mean (SD)** | **T1−Control (SE)** | **T2−Control (SE)** | **T3−Control (SE)** |
| --- | --- | --- | --- | --- |
| Household members | 5.29 (1.83) | 0.00 (0.11) | −0.04 (0.11) | 0.06 (0.11) |
| Respondent is HH head† | 0.43 (0.50) | −0.03 (0.03) | −0.01 (0.03) | 0.05* (0.03) |
| Respondent education (years)† | 6.86 (4.23) | 0.68** (0.25) | 0.24 (0.25) | 0.16 (0.24) |
| HH head education (years)† | 5.95 (4.44) | 0.67** (0.27) | 0.26 (0.27) | 0.49* (0.25) |
| Main income: employment | 0.36 (0.48) | −0.02 (0.03) | −0.03 (0.03) | 0.00 (0.03) |
| Monthly HH income (Taka) | 15,657 (9,584) | 117.8 (652.5) | 156.4 (648.2) | 961.2 (621.3) |
| Monthly health expenditure (Taka) | 1,893 (3,523) | 321.7 (218.4) | 156.1 (218.0) | 49.2 (209.4) |
| Baseline public care satisfaction† | 5.07 (1.82) | 0.08 (0.10) | 0.13 (0.10) | 0.21** (0.10) |
| Aware of public HC at baseline | 0.43 (0.49) | −0.01 (0.03) | 0.01 (0.03) | 0.02 (0.03) |

Notes: Control group means with standard deviations in parentheses. T1–T3 columns show differences from the control mean with standard errors in parentheses. † Four variables showing imbalance at the 10% level or better, included as controls in all main specifications. *** p<0.01, ** p<0.05, * p<0.10.
