# Supplementary material for: Evidence on information, access frictions, and experience in public healthcare use in rural Bangladesh: a cluster-randomized field experiment
Source: Health Policy Plan. 2026 Sep 7;41(9):1580–90. doi: 10.1093/heapol/czag109 (PMC13678817; doi:10.1093/heapol/czag109)
Supplement: Supplementary file 1 [file czag109_supplementary_data.zip › TableA3.docx]

**Table A3. Robustness Checks — Key Estimates Under Alternative Specifications**

|  | **(1) Main specification** | **(2) No baseline controls** | **(3) HC2 robust SEs** | **(4) Wild bootstrap p-value** | **(5) Excl. baseline satisfaction** |
| --- | --- | --- | --- | --- | --- |
| **Panel A: Awareness — information arm (T1)** | | | | | |
| Change in probability (95% CI) | 0.33 (0.25–0.41) | 0.35 (0.27–0.43) | 0.33 (0.25–0.41) | — | 0.33 (0.25–0.41) |
| p-value | p<0.001 | p<0.001 | p<0.001 | p=0.001 | p<0.001 |
| **Panel B: Awareness — experience arm (T3 incremental)** | | | | | |
| Change in probability (95% CI) | 0.07 (0.01–0.13) | 0.07 (0.01–0.13) | 0.07 (0.01–0.13) | — | 0.07 (0.01–0.13) |
| p-value | p=0.021 | p=0.021 | p=0.021 | p=0.032 | p=0.022 |
| **Panel C: Completed public visits — experience arm** | | | | | |
| Change in probability (95% CI) | 0.18 (0.14–0.22) | 0.19 (0.15–0.23) | 0.18 (0.14–0.22) | — | 0.17 (0.13–0.21) |
| p-value | p<0.001 | p<0.001 | p<0.001 | p<0.001 | p<0.001 |
| **Panel D: Total health expenditure — experience arm** | | | | | |
| Change in expenditure (95% CI) | −183.4 (−284–−83) | −191.2 (−294–−88) | −183.4 (−288–−79) | — | −178.2 (−279–−78) |
| p-value | p<0.001 | p<0.001 | p<0.001 | p=0.001 | p<0.001 |
| Observations | 2,261 | 2,261 | 2,261 | 2,261 | 2,261 |

Notes: Column (1) main specification (upazila fixed effects, baseline outcome, four imbalanced covariates). Column (2) omits baseline controls. Column (3) uses HC2 robust standard errors. Column (4) reports wild bootstrap p-values (999 replications). Column (5) excludes baseline public-care satisfaction from the covariate set entirely rather than adjusting for it linearly; estimates for completed visits (Panel C: 0.17 pp) and total expenditure (Panel D: −178 taka) are not materially different from the main specification, confirming that results are not driven by differential baseline satisfaction in the T3 arm. Season-of-baseline-interview controls included in all columns. 95% CIs based on cluster-robust standard errors in columns (1)–(3).
